# Antioxidant Capacity of *Potentilla paradoxa* Nutt. and Its Beneficial Effects Related to Anti-Aging in HaCaT and B16F10 Cells

**DOI:** 10.3390/plants11070873

**Published:** 2022-03-24

**Authors:** Hwa Pyoung Lee, Dong Seon Kim, Sang Hee Park, Chae Yun Shin, Jin Joo Woo, Ji Won Kim, Ren-Bo An, Changyoung Lee, Jae Youl Cho

**Affiliations:** 1 Department of Integrative Biotechnology, Sungkyunkwan University, Suwon 16419, Korea; leehwapyoung57@gmail.com (H.P.L.); wetdry20@hanmail.net (D.S.K.); lauryun@naver.com (J.W.K.); 2 Department of Biocosmetics, Sungkyunkwan University, Suwon 16419, Korea; 84701@naver.com (S.H.P.); shina8059@naver.com (C.Y.S.); wjs2jw@naver.com (J.J.W.); 3 College of Pharmacy, Yanbian University, Yanji 133002, China; anrb@ybu.edu.cn; 4 International Biological Material Research Center, Korea Research Institute of Bioscience and Biotechnology, 125 Gwahak-ro, Yuseong-gu, Daejeon 34141, Korea; changags@kribb.re.kr

**Keywords:** *Potentilla paradoxa* Nutt., antioxidant capacity, moisturizing, anti-melanogenesis, wound healing

## Abstract

Skin aging is a natural process influenced by intrinsic and extrinsic factors, and many skin anti-aging strategies have been developed. Plants from the genus *Potentilla* has been used in Europe and Asia to treat various diseases. *Potentilla paradoxa* Nutt. has been used as a traditional medicinal herb in China and has recently been shown to have anti-inflammatory effects. Despite the biological and pharmacological potential of *Potentilla paradoxa* Nutt., its skin anti-aging effects remain unclear. Therefore, this study evaluated the free radical scavenging, moisturizing, anti-melanogenic, and wound-healing effects of an ethanol extract of *Potentilla paradoxa* Nutt. (Pp-EE). Pp-EE was found to contain phenolics and flavonoids and exhibits in vitro antioxidant activities. α-Linolenic acid was found to be a major component of Pp-EE on gas chromatography-mass spectrometry. Pp-EE promoted the expression of hyaluronic acid (HA) synthesis-related enzymes and suppressed the expression of HA degradation-related enzymes in keratinocytes, so it may increase skin hydration. Pp-EE also showed inhibitory effects on the production and secretion of melanin in melanocytes. In a scratch assay, Pp-EE improved skin wound healing. Taken together, Pp-EE has several effects that may delay skin aging, suggesting its potential benefits as a natural ingredient in cosmetic or pharmaceutical products.

## 1. Introduction

Skin is the largest organ in the human body in terms of weight and extent [1]. The structure of the skin has two layers, the epidermis and the dermis, which are separated by the basement membrane. The upper layer, the epidermis, is composed of four or five layers depending on the body part: stratum basale, stratum spinosum, stratum granulosum, stratum lucidum, and stratum corneum [2]. The epidermis is a constantly renewing tissue that maintains homeostasis by balancing the loss of cells on the surface of the stratum corneum (desquamation) against the growth/migration of keratinocytes in the lower epidermis [3]. Melanocytes are present in the epidermis and hair follicles; they produce melanin, which determines the color of the skin, hair, and eyes [4]. The lower layer of skin, the dermis, consists of the papillary and reticular dermis, which are distinguished by their extracellular matrix (ECM) composition, cell type, and cell density. Beneath the reticular dermis is dermal white adipose tissue, also called the hypodermis [5]. The skin serves as a physical barrier against microbial, chemical, and mechanical hazards that can adversely affect the physiological state of the body [6].

Reactive oxygen species (ROS) are produced in almost all eukaryotic cells and regulate various physiological processes, including metabolism, proliferation, differentiation, migration, hypertrophy, and cytoskeletal dynamics. However, excessive ROS can cause oxidative damage and tissue dysfunction by altering the structural and functional properties of cellular components such as proteins, lipids, and nucleic acids [7]. Diseases for which ROS have been identified as causal factors include rheumatoid arthritis, cardiovascular disease, diabetes, neurodegenerative disorders, and cancer [8]. The free radical theory of aging holds that the accumulation of oxidative stress caused by ROS is one of the major causes of aging [9]. Antioxidants are substances that can significantly delay or inhibit the oxidation of oxidizable substrates, even when they are present in relatively low concentrations compared with the levels of oxidizable substrates [10]. Although organisms have defense systems to neutralize the oxidative effects of ROS, when there is an abnormality in the balance between ROS generation and the antioxidant system, exogenous antioxidants are needed to support the biological antioxidant system [11].

Hyaluronic acid (hyaluronan [HA]) has the ability to hold water molecules and is a key molecule in determining skin moisture. The HA content of the skin is closely associated with skin aging. The marked disappearance of epidermal HA occurs in senescent skin; thus, the epidermis loses its ability to retain water molecules, resulting in loss of skin moisture [12]. HA belongs to the glycosaminoglycan (GAG) family and is the only non-sulfated GAG. In addition, unlike other GAG family members, HA synthesis takes place in the plasma membrane, not the Golgi apparatus [13], because hyaluronan synthase (HAS), which synthesizes HA, is a membrane-bound enzyme. Mammals have three HAS isoenzymes (HAS-1, HAS-2, and HAS-3) that repeatedly add glucuronic acid (GlcA) and *N*-acetylglucosamine (GlcNAc) from the cytoplasm to the nascent polysaccharide and eventually release the elongated HA into the ECM [14]. Conversely, HA is degraded by hyaluronidase, which hydrolyzes the β-1,4-glycosidic bond between GlcA and GlcNAc. Humans have six hyaluronidase isoforms: Hyal-1, Hyal-2, Hyal-3, Hyal-4, Hyal-5, and Hyal-6 [15].

In addition to determining the color of eyes, hair, and skin, melanin plays a role in protecting the skin from ultraviolet (UV) damage [16]. Biosynthesis of melanin is induced by a variety of stimuli, including hormonal signals, UV irradiation, and inflammatory cytokines. One hormonal signal comes from α-melanocyte-stimulating hormone (α-MSH), which is released by UV-exposed keratinocytes [17]. When α-MSH binds to the melanocyte melanocortin 1 receptor, which belongs to the family of G-protein receptors, the adenylate cyclase is activated. The adenylate cyclase increases the intracellular cyclic adenosine monophosphate (cAMP) level, which activates protein kinase A (PKA) and eventually causes the phosphorylation of the cAMP response element (CREB). Activated CREB induces the expression of the *microphthalmia-associated transcription factor* (*MITF*) gene, and the phosphorylated active form of MITF acts as a transcription factor for the *tyrosinase*, *tyrosinase-related protein 1* (*TYRP-1*), and *TYRP-2* genes [18]. Tyrosinase and TYRP-1 and -2 are pivotal enzymes for melanogenesis. Tyrosinase is an enzyme that hydroxylates tyrosine to make L-3,4-dihydroxyphenylalanine, also known as levodopa (L-DOPA), a metabolic precursor of dopamine. This process is a rate-limiting step of melanin biosynthesis. Then, dopaquinone oxidized from L-DOPA becomes melanin via the action of TYRP-1 and TYRP-2 [19]. Irregular and mottled pigmentation is one of the major changes of the aging skin and is even more pronounced in sun-exposed areas. UV radiation can induce the aging of melanocytes and may severely damage melanocyte functions, leading to both hypomelanotic and hypermelanotic lesions [20].

Wound healing is also closely related to aging. As aging progresses, morphological and structural changes in the skin proceed. These changes not only affect wound healing but also make the skin more vulnerable to damage [21]. When the skin is damaged, epidermal keratinocytes proximal to the wound edge migrate to the wound site to rebuild the epidermis and restore barrier function. In that process, keratinocytes undergo various changes involved in keratinocyte activation, including cell hypertrophy, cytoarchitecture modification, the adoption of a polarized and elongated morphology, the development of cytoplasmic processes toward the wound site, altered cell adhesion, reorganization of cytoskeletal filaments, and a decrease in the number of desmosomes [22]. The stimulation of skin damage thus induces changes in the expression of various genes in keratinocytes at the wound margin. Changes in the expression of keratins are particularly notable. K1 and K10 (differentiation-specific keratins) undergo downregulation, and the expression of K6, K16, and K17 (inducible keratins) increases [23], which suggests that those keratins play a major role in preparing keratinocytes for skin regeneration. Additionally, the epidermal growth factor receptor (EGFR), a member of the receptor tyrosine kinase family, has been implicated in the skin wound-healing process. EGFR ligands are detected at high levels in wound fluid and are expressed in immune cells, follicle epithelial cells, and keratinocytes at a wound site. Immediately after wounding, EGFR and EGFR ligand expression in keratinocytes thus increases in the sweat ducts and sebaceous glands at the wound site and then decreases gradually [24]. That transient increase in expression contributes to the migration and proliferation of keratinocytes at the wound margin [25]. Several studies have reported that EGFR activation leads to increased activity of extracellular signal-regulated kinases (ERK) and protein kinase B (AKT). In proliferative and pro-survival programs of keratinocytes, the EGFR-ERK pathway plays an important role. In addition, phosphorylation of AKT led by activated EGFR improves cell survival [26]. Particularly, the wound repair system is promoted by ERK-mediated stimulation of keratinocyte migration [27].

The genus *Potentilla* is a group of flowering plants belonging to the family Rosaceae, and *Potentilla* species have been used as a traditional medicine for a long time. A Greek physician used *Potentilla erecta* (L.) Raeusch. (tormentil) to rinse oral cavity ulcerations and bathe purulent facial eczema, and in Tibet, an extract of *Potentilla anserina* was used to treat viral infections [28]. Other uses of *Potentilla* species in traditional medicine have also been documented [29]. In particular, *Potentilla paradoxa* Nutt. (also called *Potentilla supina* L.) has been used as a traditional medicinal herb in India for the treatment of astringent and febrifuge [30]. A previous study reported an ethanol extract of *Potentilla paradoxa* Nutt. could inhibit lipopolysaccharide (LPS)-induced inflammation [31]. Despite its traditional and medicinal uses, few studies have examined the anti-aging action of *Potentilla paradoxa* Nutt. on the skin. Therefore, we evaluated the effects of ethanol extract of *Potentilla paradoxa* Nutt. (Pp-EE) on anti-aging parameters, including antioxidative capacity, moisturization, anti-melanogenesis, and wound healing.

## 2. Results

### 2.1. Antioxidative Capacity of Pp-EE

Before evaluating the antioxidative activity of Pp-EE, we first checked its total phenolic content (TPC) and the total flavonoid content (TFC) to identify its potential as an antioxidant (Table 1).

**Figure 1:**
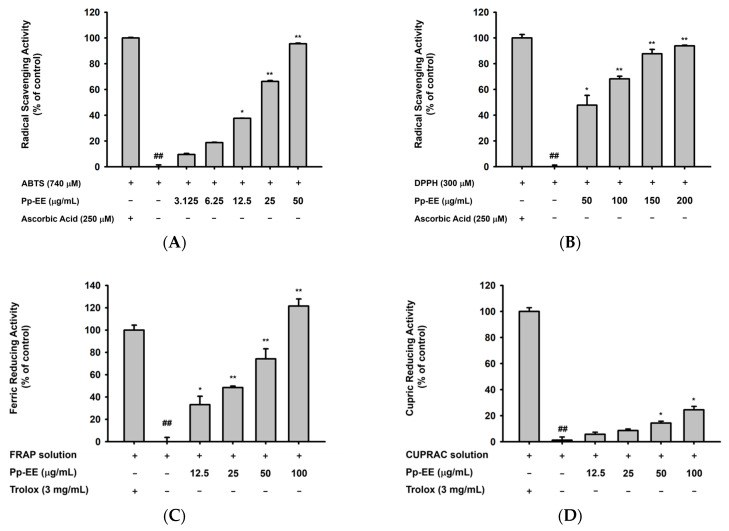

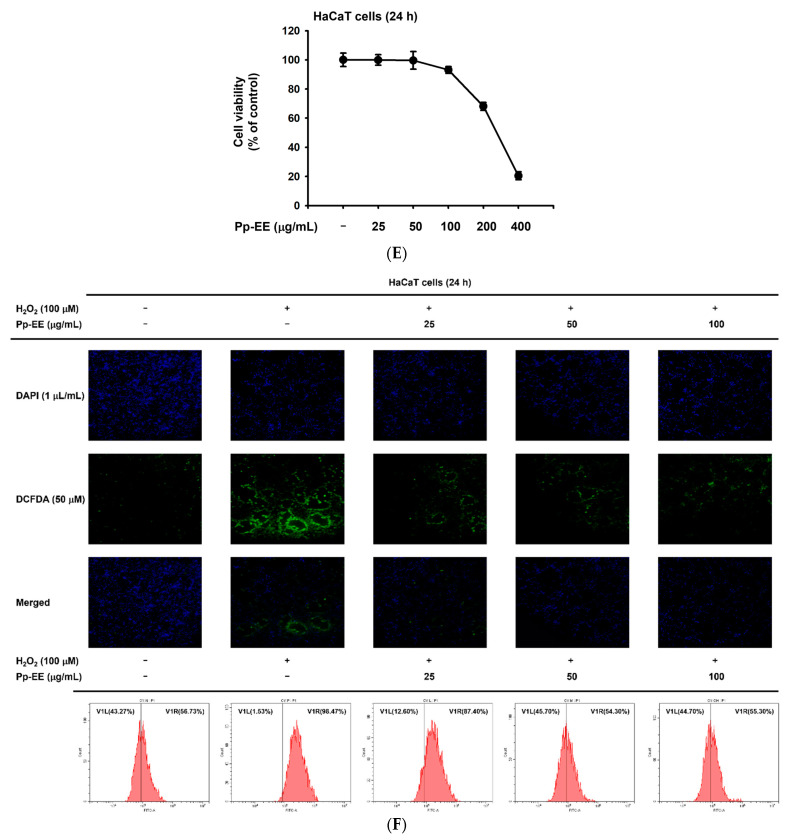
Antioxidative capacity of Pp-EE. (**A**,**B**) The radical scavenging activities of Pp-EE and ascorbic acid were evaluated using the (**A**) ABTS and (**B**) DPPH assays. (**C**,**D**) The abilities of Pp-EE and Trolox to chelate transition metals were assessed by the (**C**) FRAP and (**D**) CUPRAC assays. (**E**) The cytotoxicity of Pp-EE on HaCaT cells was evaluated using the MTT assay. (**F**) The intracellular ROS levels in HaCaT cells were detected by fluorescence microscope and flow cytometry. Results (**A**–**E**) are expressed as the mean ± standard deviation. ^##^ *p* < 0.01 compared with the positive control group, and * *p* < 0.05, ** *p* < 0.01 compared with the normal group. ABTS: 2,2′-azino-bis(3-ethylbenzthiazoline-6-sulfonic acid), DPPH: 2,2-diphenyl-1-picrylhydrazyl, FRAP: ferric reducing antioxidant power, CUPRAC: cupric ion reducing antioxidant capacity.

We also measured the radical scavenging activity of Pp-EE to determine its antioxidative capacity. First, an 2,2′-azino-bis(3-ethylbenzthiazoline-6-sulfonic acid) (ABTS) assay was conducted. ABTS oxidized by potassium persulfate or manganese dioxide produces bluish-green ABTS^•^^+^ radicals, and those radicals are decolorized upon reduction by antioxidants [32]. When Pp-EE was used at a concentration of 3.125–50 μg/mL, it exhibited dose-dependent ABTS radical scavenging activity. At 50 μg/mL, the antiradical activity of Pp-EE was similar to that of the ascorbic acid used as a positive control (Figure 1A). In the DPPH assay, stable DPPH^•^ free radicals lose their purple color upon reduction [33]. Pp-EE also scavenged DPPH^•^ radicals in a dose-dependent manner at concentrations of 50–200 μg/mL and showed significant DPPH radical scavenging activity from 50 μg/mL (Figure 1B). The ferric reducing antioxidant power (FRAP) assay uses the principle that colorless Fe^3+^–TPTZ is reduced to strongly blue Fe^2+^–TPTZ with antioxidants [34]. A total of 3 mg/mL of Trolox (Sigma, St. Louis, MO, USA) was used as the antioxidant standard [35]. We found that the ferric reducing antioxidative capacity of Pp-EE was dose-dependent (Figure 1C). The cupric ion reducing antioxidant capacity (CUPRAC) assay uses metal ions like the FRAP assay but with Cu instead of Fe. When a reducing agent reduces Cu (II) to Cu (I), the color changes from light blue to orange-yellow, which can be read at 450 nm [36]. As shown in Figure 1D, Pp-EE reduced Cu ions in a dose-dependent manner at concentrations of 12.5–100 μg/mL, although the effect was much smaller than that of the Trolox. In addition, we checked whether Pp-EE reduces ROS levels in HaCaT cells, which are immortalized human keratinocytes. First, we investigated whether Pp-EE was cytotoxic to HaCaT cells. When HaCaT cells were treated with Pp-EE for 24 h at concentrations of 25, 50, 100, 200, and 400 μg/mL, we found no cytotoxicity up to 100 μg/mL, but cell viability decreased rapidly from 200 μg/mL (Figure 1E). Therefore, we set 100 μg/mL as the maximum concentration. Intracellular ROS levels were detected visually and quantitatively by fluorescence microscopy and flow cytometry. In both cases, ROS levels were significantly reduced by Pp-EE at concentrations of 50 and 100 μg/mL (Figure 1F).

### 2.2. Phytochemical Components of Pp-EE

**Figure 2:**
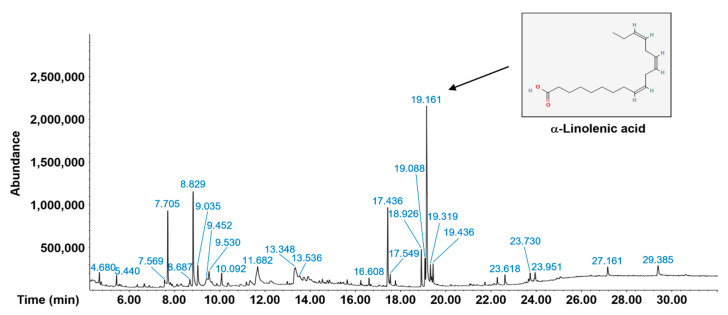
The GC-MS chromatogram of Pp-EE. GC-MS was performed as a phytochemical screen.

We also analyzed the phytochemical components of Pp-EE using gas chromatography–mass spectrometry (GC-MS). A major compound was 9,12,15-octadecatrienoic acid, (Z,Z,Z)-, also known as α-linolenic acid (Figure 2). α-Linolenic acid is a plant-based essential omega-3 polyunsaturated fatty acid. The beneficial pharmacological activities, such as anti-inflammatory, antidepressant, neuroprotective, and cardioprotective actions, of α-linolenic acid have been previously demonstrated [37,38,39,40]. In particular, it has been also found that α-linolenic acid can inhibit melanin production, showing its potential in the prevention of ultraviolet-induced skin hyperpigmentation [41]. The other compounds in Pp-EE are listed in Table 2.

### 2.3. Effects of Pp-EE on Parameters That Can Affect Skin Moisture

**Figure 3:**
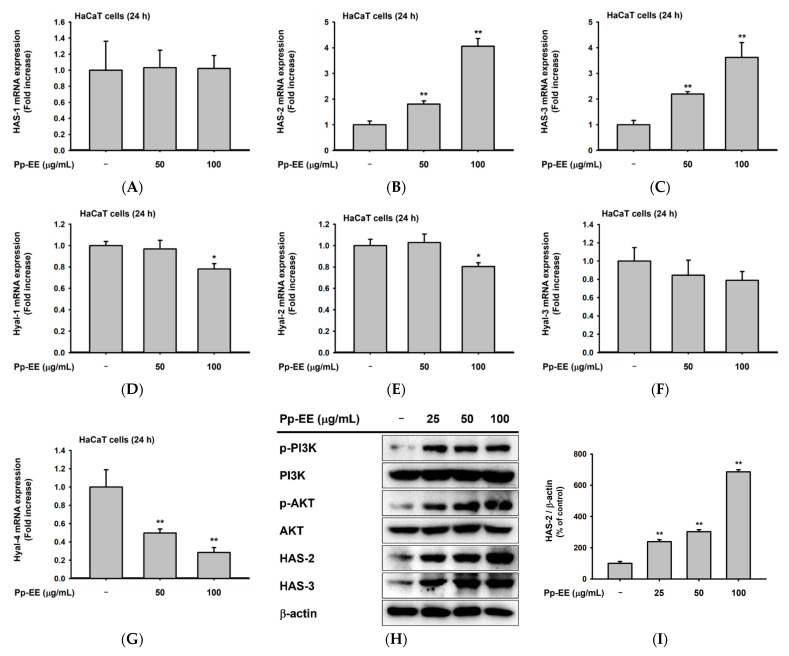

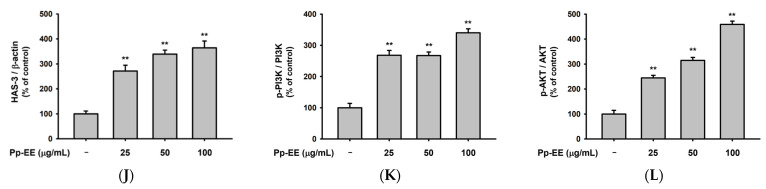
Effects of Pp-EE on parameters that can affect skin moisture. (**A**–**G**) The mRNA levels of *HAS-1*, *HAS-2*, *HAS-3*, *Hyal-1*, *Hyal-2*, *Hyal-3*, and *Hyal-4* in Pp-EE-treated HaCaT cells were determined by quantitative real-time PCR. The fold increase represents the ratio of the increased mRNA expression level of Pp-EE-treated groups to the mRNA expression level of the normal group. (**H**) The total and phosphorylated forms of PI3K, AKT, HAS-2, and HAS-3 were measured using immunoblotting analysis. (**I**–**L**) The relative intensities of the immunoblots were determined using ImageJ. All results are expressed as the mean ± standard deviation. * *p* < 0.05, ** *p* < 0.01 compared with the normal group.

We evaluated the effects of Pp-EE on parameters that are closely related to skin moisturization. HaCaT cells that produce HA were used to check the skin moisturizing effect of Pp-EE [42]. To evaluate the effect of Pp-EE on the expression of mRNA related to skin moisturize, we assessed the mRNA expression of *HAS-1*, -*2*, and -*3* after HaCaT cells were treated with 50 and 100 μg/mL of Pp-EE. The mRNA level of *HAS-1* did not change after the Pp-EE treatment, but Pp-EE increased the mRNA expression of *HAS-2* and *HAS-3* in a dose-dependent manner (Figure 3A–C). We also checked the mRNA expression of hyaluronidase (Hyal) genes. *Hyal-1*, *2-*, and -*4* were decreased by Pp-EE (Figure 3D–G). In other words, keratinocytes exposed to Pp-EE increased the expression of an enzyme that synthesizes HA and decreased the expression of an enzyme that degrades HA. *HAS-2* and *HAS-3*, which showed increased expression at the mRNA level upon Pp-EE exposure, were also investigated at the protein level. Pp-EE dose-dependently increased the protein levels of both HAS-2 and HAS-3 in HaCaT cells (Figure 3H–L). The protein level of phospho-AKT, which regulates the expression of HAS-2 [43], increased upon Pp-EE exposure, as did the protein expression of phosphorylated phosphoinositide 3-kinase (PI3K), which regulates the phosphorylation of AKT (Figure 3H,K,L).

### 2.4. Anti-Melanogenesis Activity of Pp-EE in Melanocytes

**Figure 4:**
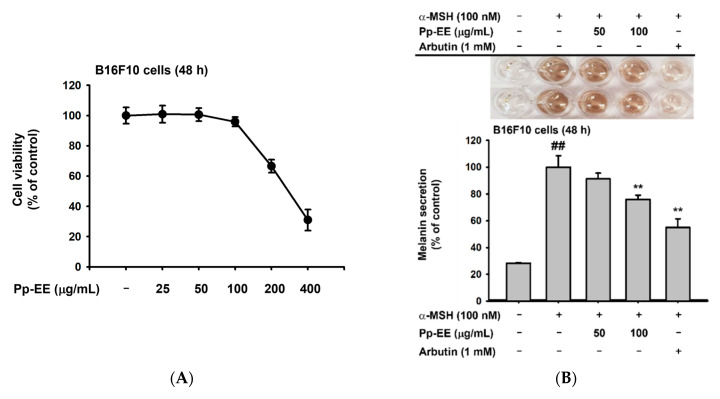

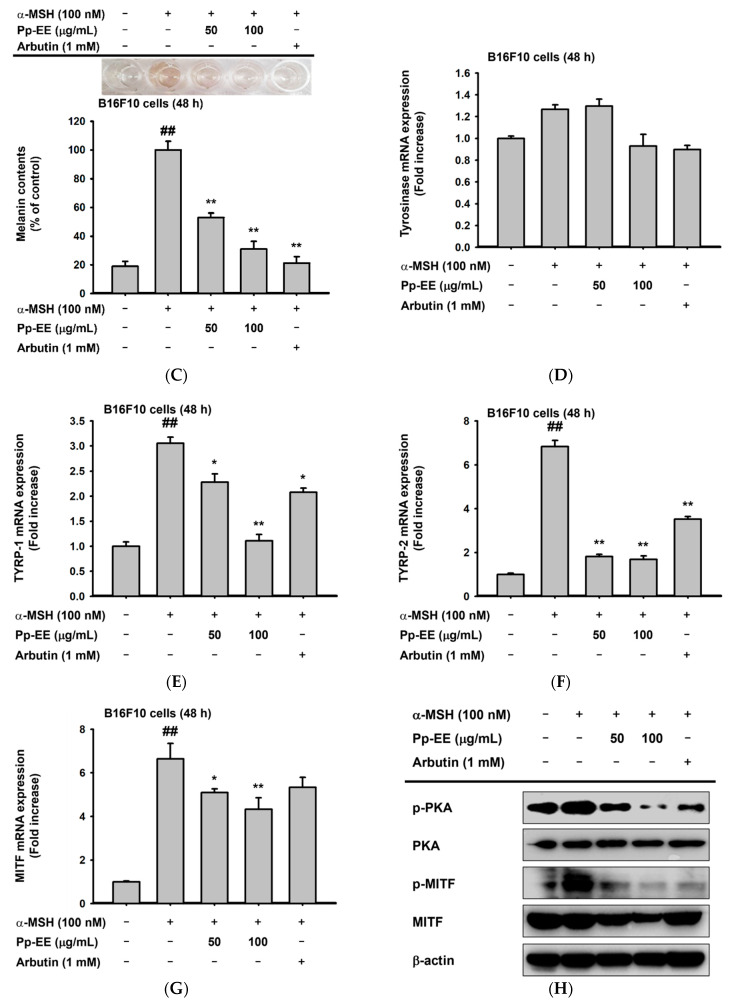

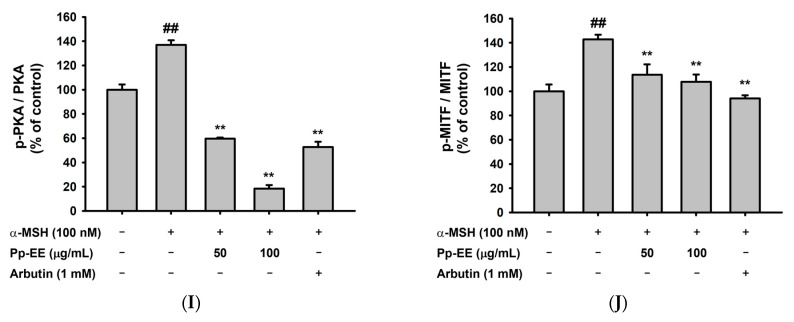
Anti-melanogenesis activity of Pp-EE in melanocytes. (**A**) The cytotoxicity of Pp-EE in B16F10 cells was assessed using the MTT assay. (**B**,**C**) The effects of Pp-EE on melanin content and secretion in B16F10 cells were evaluated using the melanin generation assay. (**D**–**G**) The mRNA levels of *MITF*, *tyrosinase*, *TYRP-1*, and *TYRP-2* were checked in B16F10 cells treated with Pp-EE or arbutin using real-time PCR. The fold increase represents the ratio of the increased mRNA expression level of α-MSH-, Pp-EE-, or arbutin-treated groups to the mRNA expression level of the normal group. (**H**) The total and phosphorylated forms of PKA and MITF were measured in an immunoblotting analysis. (**I**,**J**) The relative intensities of the immunoblots were determined using ImageJ. All the results are expressed as the mean ± standard deviation. ^##^ *p* < 0.01 compared with the normal group, and * *p* < 0.05, ** *p* < 0.01 compared with the control group.

To evaluate the potential of Pp-EE as a skin-whitening agent, we investigated whether Pp-EE could inhibit the production of melanin in B16F10 mouse melanoma cells. Again, we first checked whether Pp-EE is cytotoxic to B16F10 cells by treating them for 48 h with Pp-EE at concentrations of 25, 50, 100, 200, and 400 μg/mL. At 100 μg /mL, cell viability was 95.88 ± 3.14%, indicating no cytotoxicity, but at 200 and 400 μg/mL, cell viability fell drastically. Therefore, we set 100 μg/mL as the highest concentration (Figure 4A).

α-MSH stimulates melanocytes to induce melanogenesis. Because hyperpigmentation occurs when the biosynthesis of melanin is increased, checking a substance’s anti-melanogenic effect is one meaningful way to evaluate its whitening efficacy [44]. Arbutin is widely used as a whitening agent and suppresses melanin biosynthesis by inhibiting the activity of tyrosinase [45]. To investigate the anti-melanogenesis efficacy of Pp-EE, B16F10 cells were treated with α-MSH to induce melanogenesis and treated during 48 h with Pp-EE at concentrations of 50 and 100 μg/mL to determine whether it inhibited melanin biosynthesis. Arbutin was used as the positive control. When Pp-EE was administered, the amount of melanin secreted from the B16F10 cells was suppressed dose-dependently. Similarly, the amount of melanin in B16F10 cells also decreased dose-dependently upon Pp-EE exposure (Figure 4B,C). To understand the mechanism by which Pp-EE inhibits melanin production, we checked the mRNA expression levels of genes in the signaling pathway by which melanin is produced by α-MSH. As before, B16F10 cells were treated with α-MSH and 50 or 100 μg/mL of Pp-EE, and arbutin was used as the positive control. α-MSH treatment increased the mRNA expression of *tyrosinase* and *TYRP-1* and -*2*, which are directly involved in melanin production, and Pp-EE suppressed the increase in those mRNA expression levels except for *tyrosinase* (Figure 4D–F). The mRNA expression level of *MITF*, which regulates the expression of *tyrosinase* and *TYRP-1* and -*2*, was also upregulated by α-MSH and downregulated by Pp-EE (Figure 4G). To examine the mechanism of melanogenesis inhibition by Pp-EE at the protein level, we investigated the phosphorylation of proteins involved in melanin biosynthesis. B16F10 cells were treated with Pp-EE at a concentration of 50 or 100 μg/mL (1 mM arbutin was the positive control), and melanogenesis was induced by α-MSH. The phosphorylation of MITF, a transcription factor that plays a major role in melanin production, was inhibited by Pp-EE (Figure 4H,I). In addition, Pp-EE treatment reduced the activation of PKA, one of the main molecules in the melanogenesis pathway (Figure 4H,J).

### 2.5. Pp-EE Promotes Wound-Healing Processes in Keratinocytes

**Figure 5:**
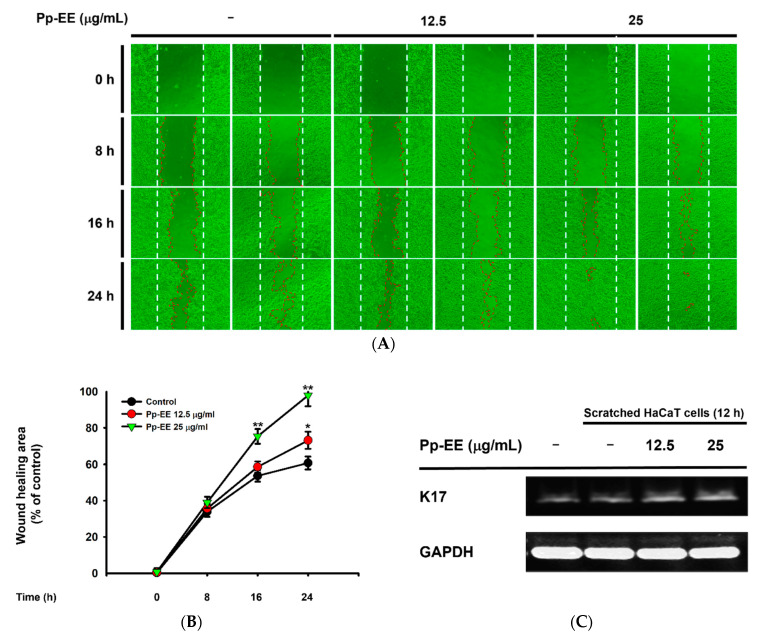

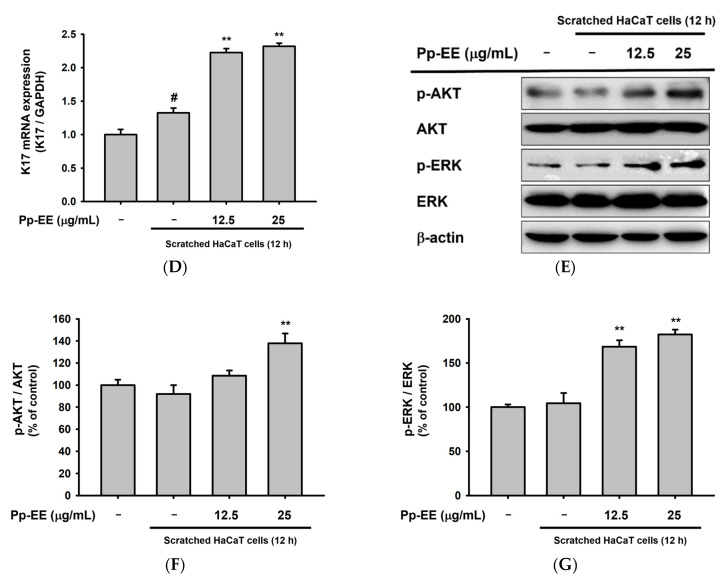
Pp-EE promotes wound-healing processes in keratinocytes. (**A**) The wound-healing activity of Pp-EE in HaCaT cells was evaluated using an in vitro scratch assay. (**B**) The scratch area closed by Pp-EE was determined using ImageJ. (**C**) Confluent HaCaT cells were scratched several times and then treated with Pp-EE (0, 12.5, and 25 μg/mL) for 12 h. The mRNA expression of *K17* was measured by RT-PCR. (**D**) The relative intensities of *K17* mRNA were determined using ImageJ. The fold increase represents the ratio of the increased mRNA expression level of scratched groups to the mRNA expression level of the normal group. (**E**) Several scratches were created on confluent HaCaT cells, which were then treated with Pp-EE (0, 12.5, and 25 μg/mL) for 12 h. The total and phosphorylated forms of AKT and ERK were measured in an immunoblotting analysis. (**F**,**G**) The relative intensities of the immunoblots were determined using ImageJ. All the results are expressed as the mean ± standard deviation. ^#^ *p* < 0.05 compared with the normal group, and * *p* < 0.05, ** *p* < 0.01 compared with the control group.

Cell migration and proliferation were investigated using a wound-healing assay to determine the ability of Pp-EE to promote healing when the skin is scratched. After making a scratch in the center of seeded HaCaT cells, Pp-EE was administered at concentrations of 12.5 and 25 μg/mL, which are concentrations that have never shown any cytotoxicity in cells (Figure 1E and Figure 4A). Then, the scratch was observed at 8 h intervals. The scratch closed much faster in the group treated with Pp-EE at 25 μg/mL than in the control group (Figure 5A,B). To investigate the wound-healing effect of Pp-EE at the gene expression level, we checked the mRNA expression of *K17*. It was upregulated by the scratch, and Pp-EE further increased the expression of this gene in a dose-dependent manner, promoting the cell migration of keratinocytes (Figure 5C,D). AKT and ERK are key downstream modulators of the EGFR signaling pathway. To determine the effect of Pp-EE on this pathway, we examined the phosphorylation levels of AKT and ERK and found that the increase in AKT and ERK activation was upregulated by Pp-EE treatment (Figure 5E–G).

## 3. Discussion

Skin aging is a process by which the skin loses its physiological function and structural integrity as a result of intrinsic and extrinsic factors [46]. Intrinsic aging of the skin is an inexorable physiological process that causes fine wrinkles, thin and dry skin, gradual dermal atrophy, etc. Extrinsic aging is caused by environmental factors such as air pollutants and UV exposure and produces coarse wrinkles and sagging and inelastic skin [47,48]. Aging of the skin cannot be avoided, but many people want to maintain youthful skin for a long time. Therefore, it could be profitable to discover new natural products that can reduce skin aging.

*Potentilla paradoxa* is now being developed as an astringent in cosmetic products with anti-aging function based on traditional knowledge passed down in the Kongju area, Chungcheongnam Province, Korea [49]. In addition, *Potentilla paradoxa* has been identified as an excellent anti-inflammatory plant through screening work performed by a nitric oxide inhibitory assay conducted with 250 plants under lipopolysaccharide-treated RAW264.7 cells (data not shown). From the deep study, we also found that expression of cyclooxygenase-2 and tumor necrosis factor-α, known as major inflammatory genes leading to skin trouble and inflammation under UV irradiation conditions [20,47], was inhibited by Pp-EE (data not shown). Due to this background information, we chose this plant over others to further examine its pharmacological activity in the skin aging process in terms of anti-oxidative activity, wound healing, skin moisturizing, and melanogenic response.

The accumulation of ROS causes skin aging [50]. Therefore, the removal of ROS that accumulates in the skin is an important process for delaying skin aging. The ABTS and DPPH radical scavenging assays and the FRAP and CUPRAC assays confirmed the abilities of Pp-EE to reduce the level of radicals. In addition, Pp-EE decreased H_2_O_2_-induced intracellular ROS levels showing the potential of Pp-EE as a new antioxidant remedy (Figure 1). Antioxidant capacity is related to the ability of flavonoids or phenolics in a plant extract to scavenge radicals [51], and the TPC and TFC assay results show that Pp-EE contains 273.26 ± 2.28 mg of gallic acid equivalent/g of extract of phenolics and 102.08 ± 2.95 mg quercetin equivalent/g of extract of flavonoids (Table 1).

It has been reported that the HA content of the skin decreases gradually as skin aging progresses [12]. A decline in HA in the skin leads to dehydration, which can cause wrinkles and abnormal skin barrier function [52]. As shown in Figure 3, Pp-EE increased the mRNA expression of *HAS*, which synthesizes HA, and suppressed the mRNA expression of *Hyal*, which degrades HA.

Skin pigmentation is one of the main characteristics of skin aging. Because melanin is closely involved in skin pigmentation, suppressing melanin formation is one way to alleviate skin pigmentation [53]. We used arbutin as a positive control in this experiment because it inhibits melanin biosynthesis by acting as a tyrosinase inhibitor [54]. Kojic acid and hydroquinone have also been used as whitening agents that work through tyrosinase inhibition, but all the tyrosinase inhibitors, including arbutin, are either ineffective in clinical practice or have unmanageable side effects, so it is necessary to find a new whitening agent to replace them [55]. Because Pp-EE inhibits the formation of melanin in melanocytes, it is worth considering it as a new whitening agent. Pp-EE suppressed the gene expression and protein phosphorylation of MITF, which is involved in the transcription of *tyrosinase* and *TYRP-1* and -*2*, and also inhibited the phosphorylation of PKA, which activates the CREB required for *MITF* gene expression. Thus, we confirmed that Pp-EE suppressed the expression of the *TYRP-1*, *TYRP-2*, and *tyrosinase* genes, thereby inhibiting melanin biosynthesis (Figure 4).

The ability to heal wounds diminishes with age in many tissues and organs. The skin is particularly vulnerable to aging-related functional deterioration, which increases susceptibility to hair loss, dryness and roughness, and impaired wound healing. As the skin ages, the proliferation of keratinocytes decreases and the migration time of keratinocytes from the basal layer to the skin surface becomes longer, which slows skin regeneration [56]. During wound healing, the wound is exposed to pathogens, and delayed re-epithelialization increases the chances of infection and chronic wound formation [57]. Therefore, a decrease in wound-healing ability due to skin aging can lead to various complications, and restoring the skin’s wound-healing ability is important [58]. The results of our wound-healing assay confirmed that Pp-EE promotes the proliferation and migration of HaCaT cells, which can help in skin scratch recovery. K6, K16, and K17 are expressed rapidly when the skin is wounded, and the expression continues until keratinocytes migrate to the wound site [59]. Pp-EE promoted the migration of keratinocytes probably by increasing the mRNA expression of *K17* in HaCaT cells. The skin wound-healing process via the EGFR signaling pathway is well understood [60]. We confirmed that Pp-EE induces the activation of AKT and ERK in the EGFR signaling pathway, thereby promoting skin wound healing (Figure 5). In addition, excessive ROS can modify or degrade ECM proteins by activating proteolysis, and they can damage dermal fibroblast and keratinocyte function, producing a detrimental effect on wound healing [61]. As shown in Figure 1, Pp-EE has an antioxidant capacity, so it can be expected to exhibit a synergic effect on skin wound healing.

Pp-EE shows promising efficacy toward all parameters related to the anti-aging activity. In chemical and cell-based assays, Pp-EE was revealed as an effective antioxidant. In addition, through in vitro experiments, it was confirmed that Pp-EE might induce moisturizing, whitening, and wound-healing promotion in the skin. These results suggest that Pp-EE can have a high potential as an active ingredient in cosmetic products. However, in order for Pp-EE to be used in cosmetics, further experiments should be performed, with dermal fibroblasts, for example. In addition, a study that aims to evaluate the photoprotective ability of Pp-EE by checking whether it can reduce skin damage caused by UV could also be a great complement/addition. 

The anti-aging capacity of Pp-EE was found to be comparable or higher than other species from the genus Potentilla in terms of its antioxidative activity (IC_50_ value = 59 μg/mL) assessed by DPPH assay (Figure 1B), although an exact comparison between these plants is difficult due to the use of different extraction methods and used plant parts [62,63]. However, the methanol extract of P. alba also exhibited lower activity than Pp-EE with an IC_50_ value of 76 μg/mL in the DPPH assay [64]. The leave’s extract of *P. rugulosa* Nakai, which shows inhibitory activity on lipid production and adipogenesis induced by endocrine-disrupting chemicals such as bisphenol A, less effectively neutralized radical generation than Pp-EE with IC_50_ value of 108 μg/mL in the DPPH assay [65]. Ethanol extract of roots of *P. atrosanguinea* also showed the capacity to reduce the DPPH radical with an IC_50_ value of 35.75 μg/mL [66]. Finally, ethanol extract of *P. atrosanguinea* aerial part showed 101.22 μg/mL as an IC_50_ value in DPPH assay [67].  Sone Potentilla species showed higher activities than Pp-EE. For example, the methanolic extract of *P. erecta* showed strong DPPH radical neutralizing activity with an IC*_50_* value of 0.012 μg/mL [68]. Antioxidative activity of *P. reptans* (aerial part) was exhibited an IC_50_ value of 12.11 μg/mL in the DPPH assay [69]. As generally known [70], the radical scavenging activity of these plants is strongly related to their content in phenolic compounds such as catechin, cinnamic acid, ferulic acid, p-coumaric acid and sinapic acid. As Table 1 shows, Pp-EE was found to exhibit 273 mg of gallic acid equivalent/g extract, superior to the value found for extracts of *P. atrosanguinea*, *P. rugulosa*, and *P. fruticosa* that revealed to have 21.75, 77.58, and 148.4 mg, respectively [65,66,71]. Considering these points, we suggest that *Potentilla* spp., in particular *P. paradoxa*, can be used as a strong anti-aging remedy with high phenolic contents linked to strong antioxidative capacity.

## 4. Materials and Methods

### 4.1. Materials

HaCaT cells (a human keratinocyte cell line) and B16F10 cells (a murine melanocyte cell line) were purchased from the American Type Culture Collection (Rockville, MD, USA). Folin and Ciocalteu’s phenol reagent, gallic acid, quercetin, aluminum chloride, 2,2′-azino-bis (3-ethylbenzothiazoline-6-sulfonic acid) diammonium salt (ABTS), potassium persulfate, ascorbic acid, 1,1-diphenyl-2 picrylhydrazyl radical (DPPH), 2,4,6-tri(2-pyridyl)-s-triazine (TPTZ), FeCl_3_·6H_2_O, dimethyl sulfoxide (DMSO), Trolox, CuCl_2_·2H_2_O, NH_4_Ac, neocuproine, (3-4,5-dimethylthiazol-2-yl)-2,5-diphenyl-tetrazolium bromide (MTT), sodium dodecyl sulfate, sodium tetraborate, carbazole, 4-hydroxyphenyl-β-D-glucopyranoside (arbutin), and α-MSH were bought from Sigma (St. Louis, MO, USA). Dulbecco’s Modified Eagle’s medium (DMEM), trypsin (0.25%), and penicillin-streptomycin solution were obtained from HyClone Laboratories (Logan, UT, USA). Fetal bovine serum (FBS) was purchased from Gibco (Grand Island, NY, USA). Additionally, 1X phosphate-buffered saline (PBS) was purchased from Samchun Pure Chemical Co. (Gyeonggi-do, Korea). TRI Reagent^®^ solution was bought from Molecular Research Center, Inc. (Cincinnati, OH, USA). The sets of primers for polymerase chain reaction (PCR) were synthetized by Macrogen (Seoul, Korea), and PCR premix was obtained from Bio-D Inc. (Seoul, Korea). Horse anti-mouse HRP-conjugated secondary antibody and goat anti-rabbit HRP-conjugated secondary antibody, the antibodies against the total and phosphorylated forms of PI3K, AKT, PKA, and ERK were purchased from Cell Signaling Technology (Beverly, MA, USA), and those against HAS-2, HAS-3, MITF, β-actin were bought from Santa Cruz Biotechnology, Inc. (Dallas, TX, USA).

### 4.2. Preparation of Pp-EE and Gas Chromatography–Mass Spectrometry

A 95% ethanol extract of the whole plant of *Potentilla paradoxa* Nutt. was procured from the International Biological Material Research Center in the Korea Research Institute of Bioscience and Biotechnology (Daejeon, Korea). The whole plant of *Potentilla paradoxa* Nutt. was collected and dried and then ground to a powder, which was used for the extraction process. The extraction was performed with 100 g of sample and 700 mL of 95% ethanol for 2 h, three times. The extract was percolated through filter paper (3 mm; Whatman PLC, Kent, UK), condensed using a rotary evaporator (Büchi AG, Flawil, Switzerland) and lyophilized using a freeze dryer (Martin Christ Gefriertrocknungsanlagen, Osterode am Harz, Germany). GC-MS analysis of Pp-EE was carried out by the Cooperative Center for Research Facilities of Sungkyunkwan University (Gyeonggi-do, Korea). Agilent 8890 GC instrument (Santa Clara, CA, USA) equipped with an Agilent J&W DB-624 Ultra Inert GC column (60 m in length × 250 μm in diameter × 1.40 μm in thickness) was used for GC. Helium gas was used as the carrier gas at a constant flow rate of 1 mL/min and the total run time of GC was 40 min. The injection volume of 1 μL was used (split ratio 20:1), and the injector temperature was maintained at 250 °C. The column oven temperature was set at 50 °C, raised 10 °C per min up to 250 °C. Agilent 5977B MSD instrument (Santa Clara, CA, USA) equipped with a detector, Series II triple-axis detector with high energy dynode and long life electron multiplier (EM), was used for MS. The scan range of MS was from 30 to 500 amu, and the solvent delay was 0.1 min. The total MS running time was 10 min. The identification of the phytochemicals was conducted by comparing the spectrum of unknown phytochemicals with the spectrum of known phytochemicals in the National Institute of Standards and Technology (NIST) library.

### 4.3. Determination of Total Phenolic Content 

The TPC of the extract was determined using Folin–Ciocalteu (FC) reagent according to the method of Singleton and Rossi Jr., with some modification [72]. This method is a colorimetric analysis in which the phenolic content of the extract turns the yellow FC reagent blue. Gallic acid was used as a reference standard. In total, 100 μL of Pp-EE (previously dissolved at a concentration of five times in distilled or deionized water) and 100 μL of 10% FC reagent were mixed with 300 μL of distilled or deionized water and incubated for 5 min at room temperature, and then 500 μL of 8% sodium carbonate and 500 μL of distilled or deionized water were added. After incubation at room temperature for 30 min, the absorbance of the reaction mixture was measured at 765 nm using a spectrophotometer (BioTek Instruments Inc., Winooski, VT, USA). All tubes containing FC reagent were protected from light. All determinations were carried out in duplicate, and the TPC is expressed as mg of gallic acid equivalent/g of extract.

### 4.4. Determination of Total Flavonoid Content

The TFC of the extract was measured according to the method described by Zhishen et al. [73], with a slight modification. Quercetin was used as the standard for the calibration curve. Pp-EE dissolved in methanol was mixed with the same volume of 2% aluminum chloride in methanol and incubated for 1 h at room temperature in the dark. After incubation, the absorbance of the reaction mixture was determined at 420 nm. Then, the TFC was calculated using a standard curve of quercetin and expressed as mg of quercetin equivalent/g of extract.

### 4.5. ABTS Radical Scavenging Activity

The ABTS radical scavenging activity of the extract was determined according to the Re et al. method [74]. This method is based on the change from the blue-green ABTS^•^^+^ to the pale blue ABTS in the presence of an antioxidant. ABTS was dissolved in distilled or deionized water to a 7 mM concentration. Then, the ABTS solution was mixed with 2.45 mM potassium persulfate in a ratio of 1:0.5 to generate ABTS^•^^+^ radical cations, which are blue-green. That ABTS^•^^+^ solution was incubated for 12–16 h at room temperature and then diluted with distilled or deionized water to yield an absorbance of 0.70 at 734 nm when diluted in half. Different concentrations of Pp-EE (3.125–50 μg/mL, previously prepared at a concentration of two times in distilled or deionized water) were mixed with the ABTS^•^^+^ solution in a ratio of 1:1, and the reaction mixtures were incubated for 15–30 min. After incubation, the absorbance of the reaction mixtures was measured at 734 nm. Ascorbic acid (250 μM) was used as the reference compound [75].

### 4.6. DPPH Radical Scavenging Activity

The protocol proposed by Brand-Williams et al. [76] was modified to determine the DPPH^•^ radical scavenging capacity of an extract. First, 300 μM of DPPH dissolved in methanol and different concentrations of Pp-EE (50–200 μg/mL, previously dissolved at a concentration of two times in methanol) were mixed in equal volume, and the absorbance at 517 nm was measured using a spectrophotometer (BioTek Instruments Inc., Winooski, VT, USA). Antioxidants reduce deep purple DPPH radicals to pale yellow DPPH or DPPH-H. The DPPH radical scavenging activity was calculated using the following equation as reported previously [77]:

DPPH radical scavenging activity % = [(A_0_ − A_1_)/A_0_] × 100 (1)

where A_0_ is the absorbance of DPPH, and A_1_ is the absorbance of the extract.

### 4.7. Ferric Reducing Antioxidant Power Assay

The FRAP assay, first known as the ferric reducing ability of plasma, was developed by Iris Benzie and J. J. Strain and modified to determine the ferric reducing power of an extract [78]. In this method, colorless TPTZ becomes blue upon reduction by an electron-donating antioxidant. First, 300 mM of acetate buffer (pH 3.6) was prepared using anhydrous sodium acetate and glacial acetic acid. Then, 10 mM TPTZ in 40 mM hydrochloric acid and FeCl_3_·6H_2_O (20 mM) were also prepared. Then, the acetic acid, TPTZ solution, and FeCl_3_ solution were mixed at a ratio of 10: 1: 1 (*v*/*v*/*v*). In a 96-well plate, 100 μL of Pp-EE solution (12.5–100 μg/mL, previously dissolved at a concentration of two times in distilled or deionized water), and 100 μL of FRAP working solution were added, mixed well, and incubated at 37 °C for 15 min in the dark. The same volume of DMSO was used as the blank control, and Trolox (3 mg/mL) was used as the positive control. The ferric reducing antioxidant power was determined by measuring the absorbance at 593 nm.

### 4.8. Cupric Ion Reducing Antioxidant Capacity Assay

The CUPRAC assay was developed by Apak et al. and slightly modified to determine the cupric reducing antioxidant capacity of an extract [79]. This method is based on the reduction of light blue bis(neocuproine) copper (II) cations [Cu (Nc)^2+^] to orange-yellow bis(neocuproine) copper (I) chelate [Cu (Nc)2^+^]. Then, 10 mM copper (II) chloride solution was prepared by dissolving CuCl_2_·2H_2_O in distilled or deionized water. NH_4_Ac (ammonium acetate) was dissolved in distilled or deionized water to make an ammonium acetate buffer (pH 7.0). Neocuproine (Nc) solution was prepared by dissolving Nc in pure EtOH at a concentration of 7.5 mM. The copper (II) chloride solution, ammonium acetate buffer, and Nc solution were mixed at a ratio of 1:1:1. Then, 200 μL of Pp-EE solution (12.5–100 μg/mL, previously prepared at a concentration of four times in distilled or deionized water) was added to 600 μL of the reaction mixture. Trolox (3 mg/mL) was used as the positive control, and an equal amount of DMSO was used as the blank control. After 1 h of incubation, the absorbance was measured at 450 nm.

### 4.9. Cell Culture

HaCaT cells and B16F10 cells were cultured in DMEM supplemented with 10% heat-inactivated FBS and 1% penicillin/streptomycin. All cells were maintained in a 5% CO_2_ incubator at 37 °C.

### 4.10. Cell Viability Assay

HaCaT and B16F10 cells were seeded in 96-well plates at a density of 4 × 10^4^ cells/mL and 6 × 10^4^ cells/mL, respectively. To evaluate the cytotoxicity of Pp-EE, all cells were treated with 25, 50, 100, 200, or 400 μg/mL of Pp-EE for 24 h. Cell viability was measured using the MTT assay [80]. After we discarded 100 μL of the cultured media, we incubated the cells with 10 µL/well of MTT solution (10 mg/mL in PBS, pH 7.4). After 3 h, the cells were treated with 100 µL of MTT stopping solution (10% sodium dodecyl sulfate with 0.01 M HCl in D.W.) overnight, and then the absorbance of the solubilized formazan at 570 nm was detected using an optical density reader (BioTek Instruments Inc., Winooski, VT, USA).

### 4.11. ROS Generation Assay

The 2′,7′-dichlorodihydrofluorescein diacetate (H_2_DCFDA), an oxidation-sensitive dye, was used to evaluate levels of intracellular ROS. HaCaT cells were seeded in 12-well plates at a density of 4 × 10^4^ cells/mL and incubated overnight. The cells were pretreated with Pp-EE (0, 25, 50, and 100 μg/mL) for 30 min and then treated with H_2_O_2_ (100 μM) for 24 h. The cells were washed with cold PBS to slow metabolism and were stained with 50 μM H_2_DCFDA for 30 min without exposure to light. The cells were fixed in formaldehyde solution (100 μL/mL) for 20 min and then washed twice with PBS, stained with DAPI (1 μL/mL) for 20 min in the dark. The fluorescence was photographed using a Nikon Eclipse Ti (Nikon, Japan) fluorescence microscope.

For flow cytometry, the cells were harvested after being treated with Pp-EE and H_2_O_2_ as above. The cells were resuspended with 300 μL of PBS with 20 μM H_2_DCFDA and then incubated for 30 min at 37 °C in the dark. The cells were washed with PBS, and the fluorescence was measured at 485/535 nm by a Beckman CytoFLEX Flow Cytometer (Beckman Coulter, Brea, CA, USA).

### 4.12. Melanin Generation Assay

B16F10 cells were seeded in 96-well plates at a density of 6 × 10^4^ cells/mL and incubated overnight. The cells were treated with Pp-EE (0, 50, and 100 μg/mL) or arbutin (1 mM) for 48 h with/without α-MSH (100 nM) [81]. To measure the amount of melanin secreted, 100 μL of cell culture medium was transferred to a 96-well plate, and the absorbance was detected at 457 nm. For the melanin content measurement, the cells were harvested with 500 μL of cold PBS and moved to e-tubes and then centrifuged to discard the PBS. The cells were lysed with 50 μL of cell lysis buffer (50 mM Tris-HCl pH 7.5, 20 mM NaF, 25 mM β-glycerophosphate pH 7.5, 120 mM NaCl, and 2% NP-40), followed by centrifugation (12,000× *g*) for 10 min. The supernatant was removed, and the pellet was dissolved with 100 μL of 1 M NaOH containing 10% DMSO at 50 °C for 30 min. Finally, 80 μL of the reactant was distributed in a 96-well plate, and the absorbance was detected at 405 nm.

### 4.13. In Vitro Scratch Assay

HaCaT cells were seeded at a density of 4 × 10^4^ cells/mL in a 6-well plate, and complete confluence was obtained after 48 h. Scratch wounds were created mechanically with a sterile pipette tip (Ø  =  0.1 mm) and uniformly made to be 0.5–0.9 mm in width, as reported previously [82]. The cells were washed with PBS to remove detached cells and debris and then treated with Pp-EE (12.5 and 50 μg/mL). Each scratched region was photographed by microscope (Nikon, Tokyo, Japan) after 0, 8, 16, and 24 h.

### 4.14. Semi-Quantitative Reverse Transcription-PCR (RT-PCR) and Quantitative Real-Time PCR (Real-Time PCR)

To evaluate the expression of genes related to skin moisture, HaCaT cells were seeded in a 12-well plate at a density of 4 × 10^4^ cells/mL and treated with Pp-EE (50 and 100 μg/mL) for 24 h. To confirm the levels of melanogenesis-related gene expression, B16F10 cells were seeded in a 12-well plate at a density of 6 × 10^4^ cells/mL, treated with Pp-EE (50 and 100 μg/mL) or arbutin (1 mM) and then treated with/without α-MSH (100 nM) for 48 h. To check the gene expression of factors related to wound healing, HaCaT cells were seeded in a 6-well plate at a density of 8 × 10^4^ cells/mL, and several artificial gaps were created on a confluent HaCaT cell monolayer using sterile pipette tips. After being washed with PBS, those HaCaT cells were treated with Pp-EE (50 and 100 μg/mL) for 24 h. Total RNA was isolated using TRI Reagent^®^ solution according to the manufacturer’s instructions. Quantification of total RNA was conducted using Take3 Micro-Volume Plate (BioTek Instruments Inc., Winooski, VT, USA). A total of 1000 ng of total RNA was used for cDNA synthesis with a cDNA synthesis kit (Thermo Fisher Scientific, Waltham, MA, USA) used according to the manufacturer’s manuals. RT-PCR and real-time PCR were conducted as previously described [83,84,85]. The primer sequences used in this experiment are listed in Table 3.

### 4.15. Preparation of Whole Cell Lysates and Immunoblotting Analysis

Pp-EE-treated HaCaT cells and Pp-EE- (or arbutin-) and α-MSH–treated B16F10 cells were collected with cold PBS using a cell scraper and then lysed for 30 min on ice in cell lysis buffer (50 mM Tris-HCL Ph 7.5, 20 mM NaF, 25 mM β-glycerol phosphate pH 7.5, 120 mM NaCl, 2% NP-40, 2 μg/mL leupeptin, 2 μg/mL aprotinin, 2 μg/mL pepstatin A, 100 μM Na_3_VO_4_, 1 mM benzamide, 100 μM PMSF, and 1.6 mM pervanadate). The cell lysates were centrifuged at 12,000× *g* for 15 min at 4 °C to settle the cell debris and then stored at −70 °C until use. Protein concentrations were determined using the Bradford assay, and then Western blotting was performed as described previously [86,87]. Briefly, samples (20 μg protein/lane) were separated by SDS-PAGE and then transferred onto PVDF membranes (Millipore, Billerica, MA, USA). After blocking with 3% BSA for 1 h at room temperature, the membranes were incubated with primary antibodies (1:2500 dilution) overnight at 4 °C. The membranes were then washed with 1X tris-buffered saline, 0.1% Tween^®^ 20 detergent (TBST) (50 mM Tris-Cl, 150 mM NaCl, 0.1% Tween-20, pH 7.5) three times for 10 min each interval, and then were incubated with the secondary antibody (1:2500 dilution) for 2 h at room temperature. After washing with TBST three times for 10 min intervals, the chemiluminescence of the membranes was detected.

### 4.16. Statistical Analysis

All data acquired from this study are presented as the mean ± standard deviation of at least three independent experiments. All the Western blots were measured using ImageJ. All results were analyzed using the Kruskal–Wallis/Mann–Whitney test. A *p*-value < 0.05 was considered statistically significant. Statistical analyses were carried out using the Statistical Package for the Social Sciences (SPSS) program.

## 5. Conclusions

**Figure 6:**
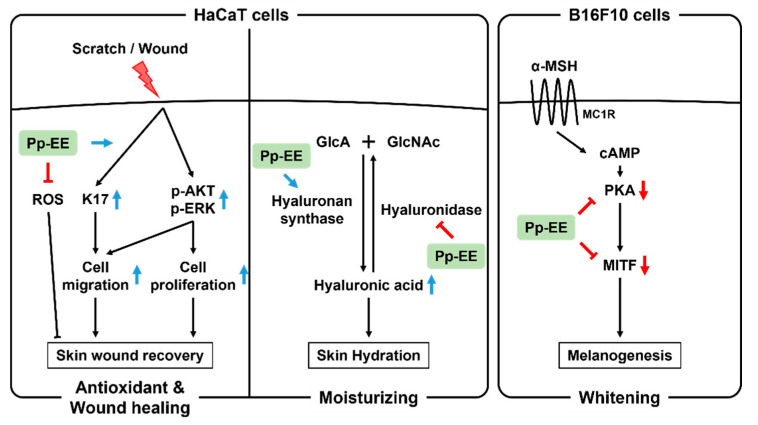
Schematic representation showing the skin moisturizing, whitening, and wound-healing effects and antioxidant capacity of Pp-EE.

Taken together, the results of this study demonstrate that Pp-EE has an antioxidant capacity, moisturizing, whitening, and wound-healing effects that may delay skin aging as summarized in Figure 6. These effects are closely related to anti-aging, suggesting that Pp-EE can be used as a raw material for cosmetics intended to inhibit skin aging.

## Figures and Tables

**Table 1:** Total phenolic and flavonoid content of Pp-EE.

Extract	TPC ^1^	TFC ^2^
Pp-EE	273.26 ± 2.28	102.08 ± 2.95

^1^ Expressed as mg of gallic acid equivalent/g of extract. ^2^ Expressed as mg of quercetin equivalent/g extract.

**Table 2:** Phytochemical analysis of ethanol extract of *Potentilla paradoxa* Nutt. by GC-MS.

Peak No.	RT ^1^	Name of the Compound	Type of the Compound	Peak Area %
1	4.680	Dimethyl sulfone	Sulfoxide	1.140
2	5.440	4H-Pyran-4-one, 2,3-dihydro-3,5-dihydroxy-6-methyl-	Pyrone	1.333
3	7.569	2-Propanamine, N-methyl-N-nitroso-	-	1.292
4	7.705	4H-Pyran-4-one, 2,3-dihydro-3,5-dihydroxy-6-methyl-	Pyrone	6.864
5	8.687	Benzofuran, 2,3-dihydro-	Benzofuran	1.332
6	8.829	5-Hydroxymethylfurfural	Alcohol	10.547
7	9.035	1,2,3-Propanetriol, 1-acetate	Glyceride	3.347
8	9.452	Methyl 4-(methylthio)butyrate	Fatty ester	2.007
9	9.530	2,4-Dihydroxy-2,5-dimethyl-3(2H)-furan-3-one	-	2.249
10	10.092	2-Methoxy-4-vinylphenol	Phenol	2.244
11	11.682	Benzaldehyde, 3-methoxy-	Benzaldehyde	8.468
12	13.348	Methyl-.beta.-D-thiogalactoside	Glucoside	6.937
13	13.536	D-erythro-Pentose, 2-deoxy-	Carbohydrate	2.028
14	16.608	1,2-Benzenedicarboxylic acid, bis(2-methylpropyl) ester	Organic acid	0.761
15	17.436	*n*-Hexadecanoic acid	Fatty Acid	8.383
16	17.549	Dibutyl phthalate	Organic acid	1.411
17	18.926	Phytol	Diterpenoid	3.241
18	19.088	9,12-Octadecadienoic acid (Z,Z)-	Fatty acid	2.894
19	19.161	9,12,15-Octadecatrienoic acid, (Z,Z,Z)-	Fatty acid	20.397
20	19.319	Octadecanoic acid	Fatty acid	3.276
21	19.436	9,12,15-Octadecatrienoic acid, ethyl ester, (Z,Z,Z)-	Fatty acid	2.564
22	22.618	Bis(2-ethylhexyl) phthalate	Organic acid	1.225
23	23.730	Linolenic acid, 2-hydroxy-1-(hydroxymethyl)ethyl ester (Z,Z,Z)-	Glyceride	1.369
24	23.951	1,2,4-Triazol-3-amine, 5-(1,3,5-trimethyl-4-pyrazolyl)amino-	Triazole	1.352
25	27.161	Vitamin E	Tocopherol	1.424
26	29.385	Hexamethylcyclotrisiloxane	Siloxane	1.913

^1^ Retention time (min).

**Table 3:** Sequences of primers used for PCR.

PCR Type	Gene Name	Sequence (5′–3′)	
RT-PCR	K17	Forward	CATGCAGGCCTTGGAGATAGA
		Reverse	CACGCAGTAGCGGTTCTCTGT
	GAPDH	Forward	CACTCACGGCAAATTCAACGGCAC
		Reverse	GACTCCACGACATACTCAGCAC
Real-time PCR	HAS-1	Forward	TGTATCCTGCATCAGCGGTC
		Reverse	GCCGGTCATCCCCAAAAGTA
	HAS-2	Forward	GTGGATGACCTACGAAGCGA
		Reverse	TACCCCGGTAGAAGAGCTGG
	HAS-3	Forward	TTGGCCTCATTCCTGTGTCC
		Reverse	CTGGCAATAAGCTGTGTAGGC
	Hyal-1	Forward	TGTGGACGTGGATGTCAGTG
		Reverse	GTAGTAGGGGTAGGTGCCCA
	Hyal-2	Forward	ATGTGCAGAACTGGGAGAGC
		Reverse	GGAAGCAAGTGTCTCGTCCA
	Hyal-3	Forward	TCTGGGCATCATAGCCAACC
		Reverse	AGAGGCCGAGTTGGTTCTTG
	Hyal-4	Forward	AACTGCATCCAAGGCCAACT
		Reverse	CTCAGCAGCTCTGGTCACAT
	MITF	Forward	TCCGTTTCTTCTGCGCTCAT
		Reverse	CTGATGGACGATGCCCTCTC
	Tyrosinase	Forward	GTCCACTCACAGGGATAGCAG
		Reverse	AGAGTCTCTGTTATGGCCGA
	TYRP-1	Forward	ATGGAACGGGAGGACAAACC
		Reverse	TCCTGACCTGGCCATTGAAC
	TYRP-2	Forward	CAGTTTCCCCGAGTCTGCAT
		Reverse	GTCTAAGGCGCCCAAGAACT
	GAPDH (Human)	Forward	GACAGTCAGCCGCATCTTCT
		Reverse	GCGCCAATACGACCAAATC
	GAPDH (Mouse)	Forward	TGTGAACGGATTTGGCCGTA
		Reverse	ACTGTGCCGTTGAATTTGCC

## Data Availability

The data are contained within the article.

## Abbreviations

Pp-ME	*Potentilla paradoxa* methanol extract
ROS	Reactive oxygen species
ECM	Extracellular matrix
EGFR	Epidermal growth factor receptor
α-MSH	α-melanocyte-stimulating hormone
ERK	extracellular signal-regulated kinases
cAMP	Cyclic adenosine monophosphate
PKA	protein kinase A
MITF	Microphthalmia-associated transcription factor
MC1R	Melanocortin 1 receptor
CREB	cAMP response element
RT-PCR	Reverse transcription-polymerase chain reaction
HPLC	High-performance liquid chromatography
FRAP	Ferric reducing antioxidant power
GC-MS	Gas chromatography–mass spectrometry
HA	Hyaluronic acid
PI3K	Phosphoinositide 3-kinase
AKT	Protein kinase B
K17	Keratin 17
CUPRAC	Cupric ion reducing antioxidant capacity
